# Production of lipopolysaccharide-induced tumour necrosis factor during influenza virus infection in mice coincides with viral replication and respiratory oxidative burst

**DOI:** 10.1155/S0962935195000299

**Published:** 1995-05

**Authors:** K. N. Masihi, H. Hintelmann, K. Madaj, G. Gast

**Affiliations:** 1 Robert Koch Institute Nordufer 20 Berlin 13353 Germany

## Abstract

Increased morbidity and mortality occur regularly during influenza epidemics. The exact mechanisms involved are not well defined but bacterial superinfection of influenza virus infected patients is considered to play an important role. In the present study, the effect of influenza virus infection on *in vivo* production of turnout necrosis factor (TNF) in response to bacterial stimuli was investigated. Release of TNF in mice infected by an aerosol of influenza virus was significant after administration of bacterial lipopolysaccharide (LPS) at 72 h, whereas administration of homologous influenza virus produced only modest amounts of TNF at 96 h. Significant production of TNF was observed 48 h after intravenous administration of infectious influenza in response to LPS but not with the homologous virus. TNF induced after influenza virus infection could be blocked by a specific murine anti-TNF monoclonal antibody. Higher TNF production following aerosol influenza infection correlated with peak titres of influenza virus in the lungs of infected mice and with enhanced generation of luminoldependent chemiluminscence.


Mediators of Inflammation 4, 181–185 (1995)

### Production of lipopolysaccharide-induced tumour necrosis factor during influenza virus infection in mice coincides with viral replication and respiratory oxidative burst

K. N. Masihi,[CA] H. Hintelmann, K. Madaj and G. Gast

Robert Koch Institute, Nordufer 20, 13353 Berlin, Germany

[CA]Corresponding Author

**INCREASED morbidity and mortality occur regularly during influenza epidemics. The exact mechanisms involved are not well defined but bacterial superinfection of influenza virus infected patients is considered to play an important role. In the present study, the effect of influenza virus infection on *in vivo* production of tumour necrosis factor (TNF) in response to bacterial stimuli was investigated. Release of TNF in mice infected by an aerosol of influenza virus was significant after administration of bacterial lipopolysaccharide (LPS) at 72 h, whereas administration of homologous influenza virus produced only modest amounts of TNF at 96 h. Significant production of TNF was observed 48 h after intravenous administration of infectious influenza in response to LPS but not with the homologous virus. TNF induced after influenza virus infection could be blocked by a specific murine anti-TNF monoclonal antibody. Higher TNF production following aerosol influenza infection correlated with peak titres of influenza virus in the lungs of infected mice and with enhanced generation of luminol-dependent chemiluminscence.**

**Key words:** Anti-TNF antibody, Chemiluminescence, Influenza virus, TNF

### Introduction

Tumour necrosis factor α (TNFα) is a pleiotropic cytokine produced mainly by macrophages. The potent immunomodulatory properties of TNF include proliferation of B[1] and T[2] lymphocytes, as well as augmentation of cytotoxic T,[3] NK cell,[4] and neutrophil[5,6] activities. In addition, TNF has been shown to synergize with other cytokines such as interferon-γ.[7] These TNF-associated host defence mechanisms can play a pivotal role in restricting the spread of microbial pathogens.

There is, however, increasing evidence that excessive and deleterious production of TNF can be triggered during severe episodes of certain infections. Raised levels of TNF and soluble TNF receptors have been detected during human immunodeficiency virus (HIV) infection in seropositive patients.[8] TNF has been associated with enhanced expression of nuclear factor kappa B, activation of HIV long terminal repeat and subsequent up-regulated transcription of the virus.[9] Production of TNF has been shown to be increased in chronic hepatitis B virus infection and clinical studies have indicated a proviral effect of TNF at higher doses.[10] TNF plays a major role also in the pathogenesis of the septic shock syndrome.[11]

Elderly persons and those with underlying health problems have more severe illness and increased complications from influenza. Additional deaths above the normal winter increase occur regularly during influenza epidemics and are used as one of the non-virologic indicators for influenza surveillance.[12] Excess mortality of at least 10 000 has been documented in each of the 19 epidemics in the United States from 1957 to 1986.[12] The exact mechanisms involved in increased morbidity and mortality during influenza infection are not as yet well defined. Bacterial superinfection of influenza virus infected patients is considered to play an important role in exacerbating virus-associated pathophysiology during influenza epidemics.[13] Influenza virus has been shown to induce the production of TNF, although the exact role of TNF during influenza infection remains unclear. Bronchoalveolar washings obtained from mice infected with influenza virus contained TNF[14] and TNF activity was demonstrated in influenza virus infected[14,15] or influenza neuraminidase treated[16] macrophages. No attempts have been undertaken to establish whether *in vivo* influenza virus infection induces production of TNF in response to bacterial stimuli. The present study was conducted to investigate *in vivo* TNF production in response to LPS in mice admin-

© 1995 Rapid Communications of Oxford Ltd istered infectious influenza by aerosol or parenteral routes.

### Materials and Methods

*Mice:* Four- to 5-week-old female NMRI mice were obtained from the animal facility maintained by the Federal Health Office.

*Pretreatment with influenza virus:* Influenza A/PR/8/34 (H1N1) was grown in the allantoic cavity of 11-day embryonated eggs using standard procedures. Infectious virus was administered to mice using a 1–2 μm diameter particle aerosol of mouse-adapted influenza virus in a Middlebrook Airborne Infection Apparatus (Tri-R Instruments, Rockville Center, USA). A 6 ml suspension of influenza virus was nebulized at 20°C using a venturi nebulizer. The $LD_{50}$ for mice was $10^{-4.5}$ by the intranasal and $10^{-1.7}$ by the aerosol route. Some groups of animals received 10 – 1 000 haemagglutinin units of infectious virus by the intravenous (i.v.) route.

*Determination of influenza virus in lungs:* Lung virus titres were determined at various time intervals after influenza aerosol infection. Lungs from five mice were removed intact, rinsed in sterile saline and quickly frozen at –70°C. After thawing, lungs were homogenized in a tissue grinder in 1 ml of cold MEM medium containing 10% fetal calf serum and antibiotics for each lung. Ten-fold dilutions of each supernatant obtained after centrifugation of the lung homogenate were inoculated in 25 μl amounts into confluent monolayers of MDCK cells in 24-well microtitre plates. Quadruplicate cultures were set up for each dilution. The virus titre was determined 2 days later by haemadsorption with chicken red blood cells. The results are expressed as $\log_{10}$ of 50% tissue culture infective doses (TCID) calculated according to the Reed and Muench method.

*Treatment with lipopolysaccharide (LPS):* Mice pretreated with influenza virus were administered 25 μg of phenol–chloroforom–petroleum ether extracted LPS from *Salmonella minnesota* (Sigma Chemical Company) by the intravenous route at various time intervals. Animals were bled from the retro–orbital plexus 1.5–2 h later and sera were stored at –20°C.

*TNF bioassay:* TNF activity in serum samples was determined by a standard fibroblast cytotoxicity assay. Briefly, 100 μl of 2.5 × $10^4$ cells per ml of a sensitive L929 cell line were added to 96-well microtitre plates (NUnc) and incubated overnight to form monolayers. Various dilutions of test samples and 1 μg of actinomycin D (Sigma Chemical Company) dissolved in Eagle's minimum essential medium were added to wells, each in a volume of 100 μl. Plates were incubated for 18 h at 37°C in the presence of 5% $CO_2$. Following incubation, the wells were aspirated and stained with 0.05% crystal violet in 20% ethanol for 15 min. After thoroughly washing the cells with distilled water, the plates were air dried. The stained cells were solubilized by adding 250 μl of ethylene glycol monomethyl ether to each well. The plates were shaken in the dark for 1 h and the optical density was read at 600 nm in a Microplate Reader MR700 spectrophotometer (Dynatech). The percentage cytotoxicity was determined. Units of TNF were calculated by plotting the regression lines of the log of the reciprocal dilution of the test sample *vs.* the percentage cytotoxicity. One unit equals the highest reciprocal of the supernatant dilution which resulted in 50% lysis.

*Treatment with anti-TNF monoclonal antibody:* Test dilutions of 1:5 serum samples in a volume of 50 μl were mixed thoroughly with the same volume of murine anti-TNF monoclonal antibody (kindly provided by BASF, Ludwighafen, Germany) containing 125 units. Test serum alone, anti-TNF alone and normal mouse serum plus anti-TNF antibody were also included in the panel. Sera were incubated for 1 h at 37°C with gentle shaking every 15 min. The plates were placed for 30 min at 4°C and the TNF assay was performed as described above.

*Chemiluminescence assay:* Aliquots of 500 μl of spleen cell suspensions in round-bottomed vials were mixed with 10 μl of luminol at a concentration of 1 mg/ml in phosphate buffered saline containing 0.4 triethylamine and incubated at 37°C for 10 min. Chemiluminescence (CL) measurements were performed at 37° C in a specially developed Biolumat model 9505 (Berthold, Wildbad, Germany) which permits simultaneous reading of six samples. After the measurement of background for 3 min, CL was generated by addition of 10 μl of particulate stimuli zymosan (Sigma) suspended in buffered saline at a concentration of 50 mg/ml. CL was continuously monitored on a programmed microcomputer.

*Statistical analysis:* Results are expressed as mean ± S. D. Statistical comparison with appropriate control groups was performed using two-tailed Student's *t*-test.

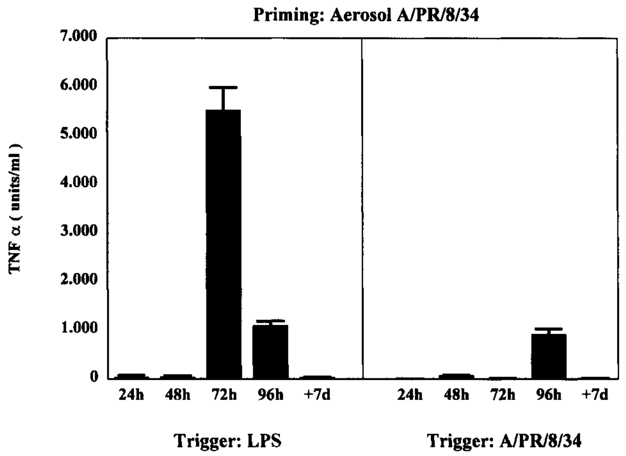

FIG. 1. Production of TNF after aerosol A/PR/8/34 influenza virus infection. LPS or homologous virus were administered intravenously as a trigger at various times after aerosol influenza infection.

### Results

*Kinetics of TNF induction in mice infected by aerosol influenza virus in response to LPS:* Animals administered an aerosol of influenza virus received an injection of LPS by the i.v. route (five mice per group) after 24, 48, 72 and 96 h, and at 7 days. Sera collected 90–120 min after LPS administration were tested for the presence of TNF activity. The results presented in Fig. 1 show significant production of TNF 72 h after influenza virus infection ($p < 0.0012$). A low, but detectable amount of TNF was produced 96 h after influenza infection ($p <$ 0.0027). When aerosol influenza infected mice were given an i.v. trigger with homologous influenza A/PR/8/34 virus instead of LPS, a modest amount of TNF was induced only at 96 h ($p <$ 0.0122) as is shown in Fig. 1. The levels of TNF production in uninfected mice after i.v. injection of LPS from *Salmonella minnesota* were of a low magnitude when compared with the 72 h-primed influenza group, and did not exceed 52 units/ml. Similar amount of LPS from virulent *Escherichia coli* can induce substantial release of TNF.

The specificity of TNF induced by influenza virus was tested using an anti-TNF monoclonal antibody. TNF induced 72 h after influenza virus infection could be blocked by a specific murine anti-TNF monoclonal antibody, as is shown in Table 1.

*Chemiluminescence response of mice infected by aerosol influenza virus:* Animals in groups of five were infected by an aerosol of influenza virus and LPS was administered by the i.v. route at 24, 48, 72, 96 h, and at 7 days. The functional activity of macrophages was assayed by zymosan-stimulated CL which was performed at the times indicated above. The results presented in Fig. 2 show that peak CL response occurred at 72 h ($p$ $< 0.0004$) followed by 96 h ($p < 0.0016$) time intervals after influenza virus infection. Influenza infected mice without LPS administration generated CL levels which did not differ significantly from those of uninfected controls (data not shown).

**Table 1.** Effect of murine anti-TNF monoclonal antibody on influenza virus induced TNF

| Serum group | Anti-TNF treatment | TNF activity (%) |
|---|---|---|
| Influenza + LPS | No | 100 |
| Influenza + LPS | Yes | 8 |
| Control | No | 0 |
| Control | Yes | 0 |
| None (saline) | Yes | 0 |

Mice infected with an aerosol of influenza virus were administered LPS by the intravenous route 72 h after infection. Serum dilutions of 1:5 were mixed with 125 units of anti-TNF monoclonal antibody, incubated and tested in a TNF bioassay.

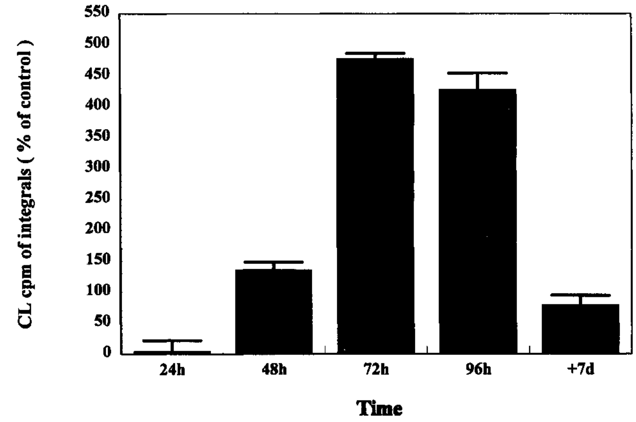

FIG. 2. Zymosan-stimulated chemiluminescence response of splenic phagocytic cells from aerosol influenza virus infected mice administered LPS by the intravenous route.

*Lung virus titres in mice infected by aerosol influenza virus:* The amount of virus present in the lungs was determined by titration of lung homogenates on confluent monolayers of MDCK cells. The results presented in Fig. 3 show the presence of detectable virus at all time periods tested. Infectivity titres from the lungs of mice infected by aerosol influenza virus but not treated with LPS were comparable to those obtained in the 24 h group (data not shown). Peak virus titres after influenza infection were detected after 72 h and were followed by the 96 h group.

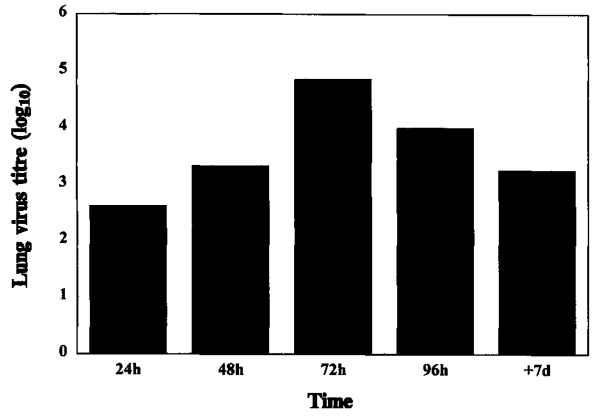

FIG. 3. Virus titres in lungs of aerosol influenza virus infected mice administered LPS by the intravenous route.

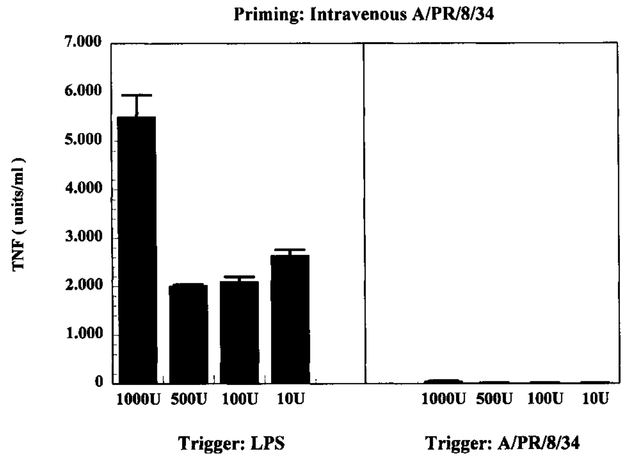

FIG. 4. Production of TNF after intravenous administration of different doses of A/PR/8/34 influenza virus. LPS or homologous virus were administered intravenously as a trigger 48 h after influenza infection.

*Kinetics of TNF induction after intravenous influenza virus infection in mice:* Animals in groups of five were injected with 10, 100, 500 or 1000 haemagglutinin units of infectious influenza virus by the i.v. route. Forty-eight h later, animals received 25 μg of LPS or 1000 haemagglutinin units of infectious A/PR/8/34 influenza virus also by the i.v. route. In preliminary experiments, the LPS trigger, administered by the i.v. route at 24, 48, 72 and 96 h, and at 7 days, induced a significant production of TNF at 48 h after i.v. influenza administration (data not shown). The results presented in Fig. 4 show induction in a dose-dependent manner of TNF. Peak TNF titres were obtained by priming with 1000 haemagglutinin units of influenza virus ($p < 0.0005$). Comparable TNF amounts were present in sera of animals receiving 500 ($p < 0.0001$) or 100 ($p < 0.0001$) haemagglutinin units of influenza virus. A trigger with homologous A/PR/34 virus was not effective in inducing TNF in A/PR/8/34 virus-primed mice.

### Discussion

Influenza virus infection can predispose the host to bacterial superinfection. Human and experimental animals infected with influenza have been shown to have increased incidence of bacterial infections due to diverse bacteria including *Streptococcus* species, *Escherichia coli* and *Haemophilus influenzae*.[13] The results of the present study demonstrate that *in vivo* influenza virus infection by the natural aerosol route can prime the host for subsequent release of TNF in response to bacterial stimuli and also extend previous *in vitro* observations showing LPS-induced potentiation of the production of TNF from influenza virus-infected macrophages.[17]

The role of TNF in viral infections is dichotomous, exhibiting both beneficial and deleterious activities. A variety of cell lines from diverse sources could be protected by TNF against infections by vesicular stomatitis virus, encephalomyocarditis virus, adenovirus and herpes simplex virus.[7,18,19] On the other hand, TNF has been shown to exacerbate several other viral infections including those caused by HIV. Primary blood monocyte-derived macrophages treated with recombinant human TNF starting before or after HIV infection enhanced viral replication of both lymphocyte-tropic and macrophage-tropic strains.[20] Recombinant TNF has been shown to activate HIV mRNA,[21] increase replication,[22] and enhance syncytium formation[23] in T-cell lines. Simian varicella virus-infected monkeys given TNF showed increased mortality,[24] and porcine monocytes treated with TNF showed an increase of African swine fever virus production.[25]

The kinetics of TNF production by the influenza virus exhibited a marked dependence on the time elapsed after influenza infection and administration of LPS. Significant TNF production was evident 72 h after aerosol virus infection. TNF can profoundly affect the functional capability of phagocytic cells, and has been shown to enhance the response of neutrophils to particulate stimuli,[5] alter surface receptors[26] and prime for enhanced production of oxygen radicals.[5,6] Administration of TNF in mice can result in an increase of the percentage of splenic macrophages.[27] In the present study, a high level of splenic cell CL activity was observed at 72 h in influenza virus-infected animals that had received LPS. It is noteworthy that the time period of enhanced CL coincides with the peak TNF production and correlated with peak titres of influenza virus which were also detected 72 h after infection.

Influenza virus or viral components have been shown to stimulate the production of interferon-$\gamma$[28] and IL-1.[16,29] In numerous studies, TNF has been shown to synergize with other cytokines including interferon-$\gamma$.[7] The intricate synergistic interaction between the putative cytokines released during the period of peak influenza virus production and resultant sequelae remain to be elucidated. The results of the present study suggests that the TNF response of the influenza virus infected host on the subsequent homologous influenza virus challenge is of a low magnitude. This could be a reflection of the protection afforded by primary influenza infection. In contrast, high levels of TNF were induced in influenza virus infected animals administered bacterial LPS. An understanding of the kinetics required for the influenza virus to sensitize a host for induction of TNF in response to bacterial stimuli may help define the critical period when complications could occur in institutionalized elderly patients and other persons at risk during influenza epidemics.

### References

1. Kehrl J, Miller A, Fauci AS. Effect of tumor necrosis factor on mitogen-activated human B cells. *J Exp Med* 1987; **166**: 786.
2. Yokota S, Geppert TD, Lipsky PE. Enhancement of antigen- and mitogen-induced human T lymphocyte proliferation by tumor necrosis factor. *J Immunol* 1988; **140**: 531.
3. Ranges GE, Figari IS, Espevik T, Palladini MA. Inhibition of cytotoxic T cell development by transforming growth factor-beta and reversal by recombinant tumor necrosis factor. *J Exp Med* 1987; **166**: 991.
4. Ostensen ME, Thiele DL, Lipsky PE. Tumor necrosis factor enhances cytolytic activity of human natural killer cells. *J Immunol* 1987; **138**: 4185.
5. Klebanoff SJ, Vada MA, Harlan JM, *et al.* Stimulation of neutrophils by tumor necrosis factor. *J Immunol* 1986; **136**: 4220–4225.
6. Ferrante A. Tumor necrosis factor alpha potentiates neutrophil antimicrobial activity. *Infect Immun* 1989; **57**: 2115–2122.
7. Feduchi E, Alonso MA, Carrasco L. Human gamma interferon and tumor necrosis factor exert a synergistic blockade on the replication of herpes simplex virus. *J Virol* 1989; **63**: 1354–1359.
8. Aukrust P, Liabakk NM, Mueller F, Lien E, Espevik T, Froland SS. Serum levels of tumor necrosis factor-alpha TNF-alpha and soluble TNF receptors in human immunodeficiency virus type 1 infection — correlations to clinical, immunologic, and virologic parameters. *J Infect Dis* 1994; **169**: 420–424.
9. Duh EJ, Maury WJ, Folks TM, Fauci AS, Rabson AB. Tumor necrosis factor-alpha activates human immunodeficiency virus type 1 through induction of nuclear factor binding to the NF-kB sites in the long terminal repeat. *Proc Natl Acad Sci USA* 1989; **86**: 5974–5978.
10. Lau JY, Sheron N, Nouri-Aria KT, Alexander GJ, Williams R. Increased tumor necrosis factor-alpha receptor number in chronic hepatitis B virus infection. *Hepatology* 1991; **14**: 44–50.
11. Tracey KJ, Cerami A. Tumor necrosis factor: an updated review of its biology. *Critical Care Med* 1993; **21**: s415–422.
12. Centers for Disease Control. Prevention and control of influenza: recommendations of the Immunization Practices Advisory Committee (ACIP). *MMWR* 1990; **39**(No. RR-7): 1–15.
13. Abramson JS, Mills EL. Depression of neutrophil function induced by viruses and its role in secondary microbial infections. *Rev Infect Dis* 1988; **10**: 326–341.
14. Vacheron F, Rudent A, Perin S, Labarre C, Quero AM, Guenounou M. Production of interleukin 1 and tumor necrosis factor activities in bronchoalveolar washings following infection of mice by influenza virus. *J Gen Virol* 1990; **71**: 477–479.
15. Sprenger H, Bacher M, Rischkowsky E, Bender A, Nain M, Gemsa D. Characterization of a high molecular weight tumor necrosis factor-a mRNA in influenza A virus-infected macrophages. *J Immunol* 1994; **152**: 280–289.
16. Houde M, Arora JS. Stimulation of tumor necrosis factor secretion by purified influenza virus neuraminidase. *Cell Immunol* 1990; **129**: 104–111.
17. Nain M, Hinder F, Gong JH, *et al.* Tumor necrosis factor production of influenza A virus-infected macrophages and potentiating effect of lipopolysaccharides. *J Immunol* 1990; **145**: 1921–1928.
18. Metan J, Digl W, Mittnacht S, *et al.* Antiviral effects of recombinant tumor necrosis factor. *Nature* 1986; **323**: 816–819.
19. Wong GHW, Goeddel DV. Tumor necrosis factor alpha and beta inhibit virus replication and synergize with interferons. *Nature* 1986; **323**: 819–822.
20. Mellors JW, Griffith BP, Oritz MA, Landry ML, Ryan JL. Tumor necrosis factor-alpha/cathectin enhances human immunodeficiency virus type 1 replication in primary macrophages. *J Infect Dis* 1991; **163**: 73–82.
21. Matsuyama T, Hamamoto Y, Soma G, Mizuno D, Yamamoto N, Kobayashi N. Cytocidal effect of tumor necrosis factor on cells chronically infected with human immunodeficiency virus (HIV): enhancement of HIV replication. *J Virol* 1989; **63**: 2504–2509.
22. Folks TM, Clouse KA, Justement J, *et al.* Tumor necrosis factor alpha induced the expression of T-cell clone. *Proc Natl Acad Sci USA* 1989; **86**: 2365–2368.
23. Vyakarnam A, McKeating J, Meager A, Beverley P. Tumor necrosis factors (alpha, beta) induced by HIV-1 in peripheral blood mononuclear cells potentiate virus replication. *AIDS* 1990: **4**: 21–27.
24. Soike KF, Czarniecki CW, Baskin CW, Blanchard J, Liggitt D. Enhancement of simian varicella virus infection in African green monkeys by recombinant human tumor necrosis factor alpha. *J Infect Dis* 1989; **159**: 331–335.
25. Esparza I, Gonazlez JC, Vinuela E. Effect of interferon-alpha, interferon-gamma and tumor necrosis factor on African swine fever virus replication in porcine monocytes and macrophages. *J Gen Virol* 1988; **69**: 2973–2980.
26. Berger M, Wetzier EM, Wallis RS. Tumor necrosis factor is the major monocyte product that increases complement receptor expression on mature human neutrophils. *Blood* 1988; **71**: 151–158.
27. Gordon C, Wofsy D. Effects of recombinant murine tumor necrosis factor on immune function. *J Immunol* 1990; **144**: 1753–1758.
28. Yamada YK, Meager A, Yamada A, Ennis FA. Human interferon alpha and gamma production by lymphocytes during the generation of influenza virus-specific cytotoxic T lymphocytes. *J Gen Virol* 1986; **67**: 2325–2334.
29. Roberts NJ, Prill JAH, Mann TN. Interleukin 1 and interleukin 1 inhibitor production by human macrophages exposed to influenza virus or respiratory syncytial virus. *J Exp Med* 1986; **163**: 511.

**Received 19 January 1995;**
**accepted in revised form 2 March 1995**
